# Yeast Fermentate-Mediated Reduction of *Salmonella* Reading and Typhimurium in an *in vitro* Turkey Cecal Culture Model

**DOI:** 10.3389/fmicb.2021.645301

**Published:** 2021-04-15

**Authors:** Kristina M. Feye, Dana K. Dittoe, Peter M. Rubinelli, Elena G. Olson, Steven C. Ricke

**Affiliations:** ^1^Center for Food Safety, Department of Food Science, University of Arkansas, Fayetteville, AR, United States; ^2^Meat Science and Animal Biologics Discovery, Animal and Dairy Sciences, University of Wisconsin-Madison, Madison, WI, United States

**Keywords:** *Salmonella*, turkey, XPC, cecal culture method, yeast fermentate

## Abstract

*Salmonella* Reading is an ongoing public health issue in the turkey industry, leading to significant morbidity in humans in the United States. Pre-harvest intervention strategies that contribute to the reduction of foodborne pathogens in food animals, such as the yeast fermentation metabolites of Original XPC^TM^ (XPC), may become the key to multi-hurdle farm to fork strategies. Therefore, we developed an anaerobic *in vitro* turkey cecal model to assess the effects of XPC on the ceca of commercial finisher tom turkeys fed diets void of XPC and antibiotics. Using the *in vitro* turkey cecal culture method, ceca were tested with and without XPC for their anti-*Salmonella* Reading and the previously defined anti-Typhimurium (ST97) effects. Ultimately, the anti-*Salmonella* effects were independent of serovar (*P* > 0.05). At 0 h post inoculation (hpi), *Salmonella* levels were equivalent between treatments at 7.3 Log_10_ CFU/mL, and at 24 hpi, counts in XPC were reduced by 5 Log_10_ CFU/mL, which was 2.1 Log_10_ lower than the control (*P* < 0.05). No differences in serovar prevalence existed (*P* > 0.05), with a 92% reduction in *Salmonella* positive XPC-treated ceca cultures by 48 hpi (*P* < 0.05). To evaluate changes to the microbiota independent of the immune response, the 16S rDNA was sequenced using the Illumina MiSeq platform. Data indicated a profound effect of time and treatment for the reduction of *Salmonella* irrespective of serovar. XPC sustained diversity metrics compared to the control, demonstrating a reduction in diversity over time (*Q* < 0.05).

## Introduction

By July 19, 2018, an outbreak of *Salmonella enterica* serovar Reading linked to ground turkey had effected 26 states in the United States. By the end of the outbreak, 358 cases were reported across 42 states and Washington DC, resulting in 133 hospitalizations and one death directly from the consumption or cross-contamination of *S.* Reading contaminated ground turkey meat (Centers for Disease Control and Prevention [CDC], 2019). In parallel, between April of 2017 and January of 2020, a total of 130 cases of *S*. Reading [British Columbia (33), Alberta (44), Saskatchewan (8), Manitoba (25), Ontario (9), Quebec (2), New Brunswick (1), Prince Edward Island (1), Northwest Territories (1), and Nunavut (6)] were confirmed across Canada (Public Health Agency of Canada [PHAC], 2021). As a result, the U.S. Food Safety and Inspection Service (FSIS), Canadian Food Inspection Agency, and other agencies across North America and the world have reached out to the turkey industry to find solutions to end this escalating public health crisis.

*Salmonella* Reading is a common serovar associated with poultry meat, as well as ground beef and pork, with a prevalence of 4.1 and 4.0% among raw swine carcasses and ground pork tested (Schlosser et al., 2000). Additionally, *S.* Reading is consistently ranked among the top 10 most isolated serovars in ground turkey, representing 25% of the 1.7% *Salmonella* positive samples (Food Safety Inspection Service [FSIS], 2014). In 2009, a survey of Oklahoma retail ground poultry meat representing five brands and six supermarket chains in Tulsa, Oklahoma, indicated that 47% (66/141) of the ground turkey meat was positive for *Salmonella* (Gad et al., 2018). Nevertheless, the incidence of foodborne illness remains relatively low by comparison. In addition to *Salmonella* and more specifically, *S*. Reading, being isolated frequently among chicken and turkey commodities, *Salmonella* isolates recovered from ground meat are phenotypically diverse, exhibiting different antimicrobial resistance and pulse-field gel electrophoresis patterns (Zhao et al., 2006). Therefore, there is a critical need for further interventions at the farm and processing levels in order to reduce the incidence of *S.* Reading and other *S. enterica* serovars.

Original XPC^TM^ is a concentrated *Saccharomyces cerevisiae* fermentate that is added to animal feed to maximize the nutritional benefits (Diamond V Original XPC^TM^ (XPC), 2020). In addition to extensive nutritional characteristics, fermentation increases the presence of lactic acid bacteria, promotes basic pH, and produces high amounts of organic acids which potentially help sustain the feed from pathogen colonization prior to ingestion and promote a good gastrointestinal environment in animals (Canibe and Jenses, 2012). The fermentation metabolites of XPC have demonstrated anti-*Salmonella* effects against multiple *Salmonella* serovars (Feye et al., 2016a,b; Rubinelli et al., 2016; Park et al., 2017; Roto et al., 2017). These effects include the reduction of multimodal antibiotic resistance and attenuating the virulence of the pathogen, specifically (Feye et al., 2019). A likely mechanism associated with XPC is rooted in the demonstrated changes to the microbiota in response to XPC supplementation, shifting the metabolites present, and ultimately reducing *Salmonella* fitness (Park et al., 2017; Rubinelli et al., 2016; Feye et al., 2019). The same mechanism likely drives the immune-modulating response that reduces peripheral inflammation while increasing the phagocytosis of *Salmonella* (Feye et al., 2016b, 2019; Nelson et al., 2020).

Due to the significance of *S.* Reading outbreaks and the previously demonstrated anti-*Salmonella* effects of XPC, it is the objective of the current study to determine whether the presence of XPC could lead to the reduction of a nalidixic acid-resistant (64 μg/mL) strain of *S.* Reading in an *in vitro* turkey cecal culture model. The *in vitro* fermentation cecal model is a robust method to research microbial interactions while avoiding host factors, such as an immune response (Feye et al., 2019; Poeker et al., 2019). As this model was untested in turkeys, a previous strain of nalidixic acid-resistant *Salmonella* Typhimurium that was successfully reduced by XPC in the *in vitro* chicken cecal model was used as a control. Therefore, if *S*. Reading and Typhimurium were reduced equally or at least followed the same trend, the model should represent the cecal microbial ecology and its response to the presence of XPC.

## Materials and Methods

### Tom Turkey Ceca Procurement

There were two independent trials for this experiment, with five ceca being collected at each trial from a USDA inspected commercial processing plant located in Arkansas, United States, and transported to the Center for Food Safety at the University of Arkansas on ice (*n* = 10). The turkeys were processed as a component of standard processing production practices by a commercial collaborator and were exempt from the Institutional Animal Care and Use Committee. The ceca were aseptically collected by commercial collaborators and were at the laboratory within one hour of slaughter for use in the experiment.

### *In vitro* Tom Turkey Cecal Culture Model

**FIGURE 1:**
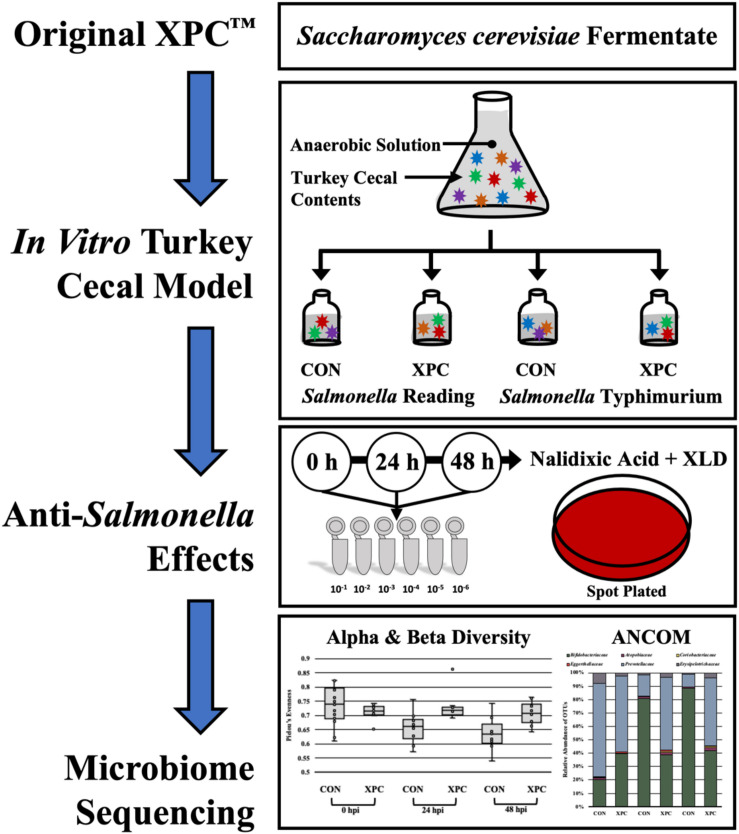
Experimental design of the anaerobic *in vitro* turkey cecal cultures.

The cecal cultures were prepared in an anaerobic chamber (7% H_2_, 5% CO_2_, and 88% N_2_) to closely mimic the cecal environment (Rubinelli et al., 2016; Figure 1). Once introduced to the anaerobic chamber, a 0.1 g portion of ceca contents from the proximal end of the ceca inferior to the ileal–cecal junction was cut, and the contents were aliquoted into sterile 1.5 mL microcentrifuge tubes. Cecal contents were subsequently re-suspended in 1 mL of Anaerobic Dilution Solution (ADS). The ADS medium consisted of 0.45 g/L K_2_ HPO_4_, 0.45 g/L KH_2_PO_4_, (NH_2_)_2_SO_4_, 0.9 g/L NaCl, 0.1875 g/L MgSO_4_⋅7H_2_O, 0.12 g/L CaCl_2_⋅2H_2_O, 1 mL of 0.1% resazurin, and 0.05% v/v cysteine HCl and was autoclaved. Filter sterilized 0.4% CO_2_ was added post autoclaving (Rubinelli et al., 2016). Prior to the start of the experiment, the ADS was allowed to become anaerobic. As the blue resazurin dye became clear, it indicated minimal oxygen present in the system.

Approximately, 100 mg of cecal content was resuspended in 900 μL of ADS and vortexed until completely homogenized. Following, a 1:3000 dilution was performed by adding 1 mL of the re-suspended ceca into sterile Erlenmeyer flasks containing 299 mL ADS each, and the cecal cultures were homogenized by pipetting. A 20 mL portion from each flask was then aliquoted to each of four serum bottles containing the following treatments: (1) 0.25 g of the basal diet feed inoculated with *S*. Typhimurium; (2) 0.25 g of the basal diet inoculated with *S*. Reading; (3) 0.25 g of the basal diet + 0.2 g XPC (1.25% w/v) inoculated with *S*. Typhimurium; and (4) 0.25 g of the basal diet + 0.2 g XPC (1.25% w/v) inoculated with *S*. Reading. The basal diet used in the current study was formulated to meet the recommend nutrient requirements for broilers according to the NRC guidelines (Table 1; National Research Council [NRC], 1994). Although chicken feed was utilized in the current study, the metabolizable energy and protein composition of the basal diet was comparative to standard commercial turkey diets (National Research Council [NRC], 1994). The XPC was a fresh shipment from Diamond V and was received as a standard representation of their commercial production and was visually free of mold, insects, or other contaminants.

**TABLE 1:** Composition of the basal broiler diet used in this study.

Ingredient^1,2^	(%)
Corn	59.36
Soybean meal	32.85
Meat and bone meal (50%)	2.50
Poultry oil	2.01
Sodium chloride	0.38
Sodium bicarbonate	0.00
Limestone	0.80
Dicalcium phosphate	1.13
Vitamin premix	0.10
Mineral premix	0.10
Choline chloride	0.10
Selenium premix (0.6%)	0.02
Santoquin	0.02
L-Lysine HCL	0.17
DL-Methionine	0.30
L-Threonine	0.11
Copper chloride	0.02
Xylanase	0.01
Phytase	0.02
Total	100.00

*^1^Ingredient nutrient composition was analyzed before formulating the diet.*

*^2^Metabolizable energy (kcal/kg) was 2977.*

Once the ADS solution was clear, and therefore anaerobic, the bottles were subsequently sealed with a thick rubber stopper and crimped to retain the anaerobic headspace. The serum bottles were pre-incubated at 42°C and oscillated at 130 rpm for 24 h. Pre-incubation was chosen as previously published studies indicate that XPC is effective after it has been metabolized by the cecal contents (Rubinelli et al., 2016).

### *Salmonella* Inocula Preparation and Inoculation

*Salmonella* Reading used in the current study was provided by Diamond V (Cedar Rapids, IA, United States), where the company obtained the donated isolate during the *S.* Reading outbreak among raw turkey products in the United States (Centers for Disease Control and Prevention [CDC], 2019). The *S.* Typhimurium (ST97) strain used in the study was isolated from chicken by the Poultry Health Laboratory at the University of Arkansas (Park et al., 2017). Both *S*. Reading and Typhimurium were resistant to nalidixic acid at 64 μg/mL prior to obtaining the cultures from Diamond V and the Poultry Health Laboratory. In order to use both *S*. Reading and Typhimurium as marker strains throughout the study, significant care was taken to ensure that selective pressure (64 μg/mL of nalidixic acid) was maintained throughout the culture phase before use and during stock preparations. Before initiating the experiment, frozen stocks of *S.* Reading and Typhimurium (ST97) from the Center for Food Safety’s culture collection were inoculated into tryptic soy broth (TSB; BD Biosciences, San Jose, CA, United States). Cultures were prepared in an oscillating incubator (125 rpm) aerobically at 37°C for 12–16 h. Immediately following, cultures were streaked onto Xylose Lysine Deoxycholate (XLD, HiMedia, West Chester, PA, United States) containing 64 μg/mL of nalidixic acid (Alfa Aesar, Haverhill, MA, United States) and incubated in an oscillating incubator (125 rpm) overnight (12–16 h) at 37°C, aerobically. One colony was used to inoculate 35 mL of TSB and incubated overnight under the aforementioned conditions. The inoculum was prepared as previously described (Rubinelli et al., 2016).

After the pre-incubation of the turkey cecal cultures with and without XPC, cultures were inoculated with the marker strains of *S*. Reading or Typhimurium according to Rubinelli et al. (2016). As such, cultures designated as the *Salmonella* treatment bottles were inoculated with one of the two serovars of *Salmonella*, with 10^6^ cells/mL *S*. Typhimurium ST97 or 10^6^ cells/mL *S*. Reading. After inoculation and the zero timepoint enumeration, serum bottles were sealed with a thick rubber stopper and crimped to retain the anaerobic headspace.

### *Salmonella* Enumeration

As the tom turkey cecal cultures were inoculated with *S*. Reading and Typhimurium, a 1 mL aliquot was taken for the zero timepoint enumeration. The bottles were then resealed with the thick rubber stoppers and crimped inside of the anaerobic chamber to retain the anaerobic headspace. Cultures were then placed into the oscillating incubator at 42°C and oscillated at 130 rpm until the 24 and 48 h timepoints occurred where they were subsampled under anaerobic conditions and plated. At 0, 24, and 48 h, the aliquots were serially diluted (1:10) with 1× phosphate buffered saline (PBS). Tom turkey cecal cultures were spot plated on XLD agar containing 20 μg/mL nalidixic acid in duplicate and allowed to dry completely prior to inverting the plates (Jett et al., 1997; Herigstad et al., 2001; Naghili et al., 2013). The plates were incubated at 37°C for 24 h, and colonies were enumerated. Prevalence was identified by positive plates using the spot plate method, not by enrichment. Therefore, while the term prevalence was used throughout the study, it is not a true measure of prevalence but that the colonies were not detected. The necessary caveats should be considered when evaluating data presented herein as traditional enrichment may yield different results.

The input strain was confirmed via an antibiogram as the *Salmonella* strains were 64 μg/mL resistant to nalidixic acid. Briefly, *Salmonella* isolated from XLD media containing 20 μg/mL were individually isolated into a 96-well flat-bottom plate containing 200 μL of sterile, pre-warmed TSB. The plate was incubated statically for 24 h at 37°C and pin replicated into individual plates containing nalidixic acid at 64 μg/mL. The concentration of nalidixic acid (64 μg/mL) was the chosen resistance level provided by Diamond V Mills. All colonies enumerated throughout this study were subjected to this antibiogram. Irrespective of turbidity, turbid wells were considered positive for resistance. Additionally, non-inoculated controls were included to control for environmental contamination.

### DNA Extraction and Microbiome Sequencing

All timepoints were collected for microbiome analysis and stored in 1.5 mL microcentrifuge tubes at −20°C until they could be processed. Individual aliquots of each sample (200 mg) were extracted using the Fast Mini Stool Kit (Qiagen, Hilden, Germany) using standard protocols and subsequently diluted to 10 ng/μL using a Nanodrop^TM^ 1000 spectrophotometer (Thermo Scientific, Waltham, MA, United States). Following the Kozich et al. (2013) protocol, 16S rDNA gene sequencing libraries targeting the V4 region were prepared using a high-fidelity polymerase, Pfx, under the recommended PCR conditions (Invitrogen, Carlsbad, CA, United States). Libraries were then quantified with the KAPA Library quantification kit (Kapa Biosystems, Inc., Wilmington, MA, United States) specified for Illumina platforms and with a broad range dsDNA kit for a Qubit fluorometer (Invitrogen, Carlsbad, CA, United States). Library amplicon size in base pairs (bp) was determined using an Agilent bioanalyzer (Agilent Technologies, Santa Clara, CA, United States). The library was then diluted to 20 pM with HT1 buffer and 10% PhiX, loaded into a Miseq V2 cartridge, and sequenced on an Illumina MiSeq as per standard Illumina practices (Illumina, San Diego, CA, United States). Resulting sequences (fastq files) were downloaded from Illumina BaseSpace and uploaded to a GitHub depository^1^. Data were also uploaded to NCBI Sequence Read Archive (SRA) under the accession PRJNA716729.

### Bioinformatic Analyses

Although a total of 10 cecal cultures were sampled directly for microbiome analysis at each timepoint and inocula (*n* = 10), eight samples per treatment and timepoint passed quality control measures prior to sequencing. Therefore, only eight replications per treatment and inocula are represented in the microbiome data.

The subsequent data were downloaded from Illumina BaseSpace, de-multiplexed, and locally uploaded into QIIME2.2019.10 (Bolyen et al., 2018). Sequences were filtered for quality and trimmed via DADA2, with chimeras filtered by consensus and quality (Callahan et al., 2016) (via q2−dada2). A contingency filter removed frequencies of less than 3 per sample to reduce noise. The phylogenetic trees were created in mafft (Katoh et al., 2002) (via q2−alignment), with the taxonomical alignments aligned to SILVA full OTU sequence with a confidence limit of 95% (Bokulich et al., 2018). Alpha and Beta diversity analyses were rarified at 8000 reads, which was when the read samples reached their maximum and sustained differences in diversity. Alpha diversity was assessed for richness with the Shannon diversity index and evenness using Pielou’s Evenness. Beta-diversity metrics were analyzed with quantitative indices, such as Jaccard dissimilarity index and weighted UniFrac distance matrix (Lozupone et al., 2007), using the Analysis of Similarity (ANOSIM) function, which takes into account dispersion and the average variation of the populations (Anderson, 2001). Significant features of time were plotted along the *X*-axis and visualized using the Emperor PCoA plot. The Alpha diversity analytics included the Kruskal–Wallis tests for pairwise differences in the treatment groups (Kruskal and Wallis, 1952). The differential abundance was identified using the ANCOM analysis, which was chosen due to the low sample size (Mandal et al., 2015).

### Statistical Analyses

Per sample tested, the arithmetic mean colony-forming unit counts of the two technical replicates per biological replicate and treatment were taken and imported into Excel (Microsoft, Redmond, CA, United States). The colony-forming unit counts were Log_10_ transformed, and the document was exported into SAS statistical software (Version 9.4, SAS Institute Inc., Cary, NC, United States). All zeros were assumed zero throughout this trial as they were below the limit of detection. *Salmonella* CFU data were analyzed for the main effects (Treatment, Serovar, Time) and evaluated with a full factorial interaction with Time as a repeated measure. Means were separated using least squares means (LSMEANS) with the PDIFF option. Prevalence was a binomial response defined as positive (growth) or negative (no growth) and analyzed with a χ^2^ analysis using the Nominal Logistic function. Significance was defined at *P* ≤ 0.05.

Microbiome main effects were considered significant if the main effect had *P* ≤ 0.05, and the pairwise effect had *Q* ≤ 0.05 with each statistical measurement within the QIIME2 pipeline. The *Q*-value represents the *P*-value as adjusted for a strict false discovery rate, which is already incorporated into the QIIME2.2019.10 pipeline. All microbiome analyses were done in accordance with the QIIME2 pipeline and aforementioned packages defined in the previous method section.

## Results

### XPC Exhibits Reduced *Salmonella* Reading Load

*Salmonella* Reading was evaluated alongside *S.* Typhimurium to determine if the reduction of *Salmonella* was serovar specific. The preadaptation step was taken to ensure that the metabolites of XPC were properly liberated by the microbiota (Rubinelli et al., 2016). Samples from the turkey cecal cultures were taken immediately after inoculation 0 h (s) post-inoculation (hpi), 24 hpi, and 48 hpi (*n* = 10, *N* = 160, *k* = 4, 3 timepoints). There were no differences in the prevalence of the *Salmonella* between the two serovars utilized in this study (*P* = 0.7735, Supplementary Tables 1, 2). As there was no significant serovar effect, the serovars were pooled to create a protected pool (*n* = 20, *N* = 160). Using Time as a repeated measure, the effect of time and treatment, and their subsequent interaction was significant (*P* < 0.0001). Therefore, as time progressed, there was a decrease in *Salmonella* prevalence (Main Effect of Time, *P* < 0.0001; Table 2). There was an 11% reduction of *Salmonella* prevalence at 24 hpi and a 61% reduction by 48 hpi. As for the effect of treatment (Main Effect of Treatment, *P* < 0.001), the addition of XPC reduced *Salmonella* prevalence by 25% compared to the control (Table 1). There was a 92% drop in *Salmonella* prevalence over time (0–48 h hpi) in the XPC-treated turkey cecal cultures (Treatment × Time Interaction, *P* < 0.0001; Table 2). In the control turkey cecal cultures, there was a 30% decrease in the prevalence of *Salmonella* over time (0–48 hpi). At 48 hpi, the control turkey cecal cultures had a prevalence of 70% where XPC-treated cultures had a 7.58% *Salmonella* prevalence.

**TABLE 2:** The main effect of time, treatment, and the interaction of treatment × time on the prevalence of overall *Salmonella* when inoculated into an *in vitro* turkey cecal model with and without Original XPC^TM^ at 0, 24, and 48 h post inoculation (hpi) over two trials.

Time^1^		*P*-value	<	0.0001
Variable		Mean^2^ (%)		SEM
0		100.00^a^	±	0.00
24		89.95^a^	±	15.00
48		38.79^b^	±	15.63

**Treatment^3^**		***P*-value**	**<**	**0.0001**
**Variable**		**Mean^4^ (%)**		**SEM**

CON		89.16^a^	±	10.15
XPC		63.33^b^	±	15.23

**Time × Treatment^5^**		***P*-value**	**<**	**0.0001**
**Variable**		**Mean^6^ (%)**		**SEM**

0	CON	100.00^a^	±	0.00
	XPC	100.00^a^	±	0.00
24	CON	97.47^a^	±	6.89
	XPC	82.42^ab^	±	13.69
48	CON	70.00^b^	±	14.49
	XPC	7.58^c^	±	11.29

*^1^Main effect of time: *P* < 0.0001, *n* = 40, *N* = 120, *k* = 3, Pooled SEM = 4.79.*

*^2^Those with different superscripts (a–b) are significantly different from one another (*P* < 0.05).*

*^3^Main effect of treatment: *P* < 0.0001, *n* = 60, *N* = 120, *k* = 2, Pooled SEM = 3.86.*

*^4^Those with different superscripts (a–b) are significantly different from one another (*P* < 0.05).*

*^5^Interaction of Treatment and Time: *P* < 0.0001, *n* = 20, *N* = 120, *k* = 6, Pooled SEM = 6.69.*

*^6^Those with different superscripts (a–c) are significantly different from one another (*P* < 0.05).*

*There was a trend in the effect of *Salmonella* serovars (*P* = 0.07, *n* = 60, *N* = 120).*

Using time as a repeated measure, the main effect of serovar was not significant throughout this study for any analyses (*P* = 0.7779, Supplementary Tables 3, 4). However, the main effects of time and treatment, as well as their interaction (treatment × time), were significant (*P* < 0.01; Table 3). Pairwise contrasts were performed to examine the interactions at each timepoint. Over time (Main Effect of Time, *P* < 0.0001), *Salmonella* was reduced from 7.32 to 3.20 and from 3.20 to 1.19 Log_10_ CFU/mL from 0 to 48 hpi. With the main effect of treatment, turkey cecal cultures treated with XPC resulted in lower concentrations of *Salmonella* compared to those not treated, 3.26 and 4.55 Log_10_ CFU/mL, respectively (Main Effect of Treatment, *P* < 0.0001). Overall, the interaction of treatment and time (Treatment × Time Interaction, *P* < 0.0001, Table 3) resulted in a stepwise decrease in *Salmonella* load in the CON and XPC groups. However, there was no difference between the XPC or control group at the start of the trial. At 24 hpi, there was a 3 Log_10_ CFU/mL (7.31–4.22 Log_10_ CFU/mL) reduction in the control group versus a 5 Log_10_ reduction (7.33–2.19 Log_10_ CFU/mL) of *Salmonella* in the XPC-treated group. By 48 h, the XPC-treated group had almost reduced *Salmonella* completely as compared to the control, 0.25 and 2.12 Log_10_ CFU/mL of ceca culture, respectively. The un-inoculated controls remained negative throughout the time frame of the course of the study. All *Salmonella* isolated were confirmed to be resistant to 64 μg/mL nalidixic acid through an antibiogram; therefore, the plate concentration did not pick up any additional native *Salmonella* with the 20 μg/mL resistance profile.

**TABLE 3:** The effect of time and treatment and subsequent interaction on the total load of *Salmonella* at 0, 24, and 48 h post-inoculation (hpi) over two trials where *Salmonella* Typhimurium and *S*. Reading were inoculated into an *in vitro* turkey cecal model with and without Original XPC^TM^.

Time^1^		*P*-value		<	0.0001
Variable		Mean^2^ (Log_10_ CFU/mL)			SEM
0		7.32	^a^	±	0.026
24		3.20	^b^	±	0.276
48		1.19	^c^	±	0.261

**Treatment^3^**		***P*-value**		**<**	**0.0001**
**Variable**		**Mean^4^ (Log_10_ CFU/mL)**			**SEM**

CON		4.55	^a^	±	0.406
XPC		3.26	^b^	±	0.326

Time*Treatment^5^		***P*-value**		**=**	**0.0012**
**Variable**		**Mean^6^ (Log_10_ CFU/mL)**			**SEM**

0	CON	7.31	^a^	±	0.038
	XPC	7.33	^a^	±	0.037
24	CON	4.22	^b^	±	0.326
	XPC	2.19	^c^	±	0.293
48	CON	2.12	^c^	±	0.398
	XPC	0.25	^d^	±	0.198

*^1^Main effect of time: *P* < 0.0001, *n* = 40, *N* = 120, *k* = 3, Pooled SEM = 0.21.*

*^2^Those with different superscripts (a–c) are significantly different from one another (*P* < 0.05).*

*^3^Main effect of treatment: *P* < 0.0001, *n* = 60, *N* = 120, *k* = 2, Pooled SEM = 0.17.*

*^4^Those with different superscripts (a–b) are significantly different from one another (*P* < 0.05).*

*^5^Interaction of Treatment and Time: *P* = 0.0012, *n* = 20, *N* = 120, *k* = 6, Pooled SEM = 0.29.*

*^6^Those with different superscripts (a–d) are significantly different from one another (*P* < 0.05).*

### Microbiota Analysis

**FIGURE 2:**
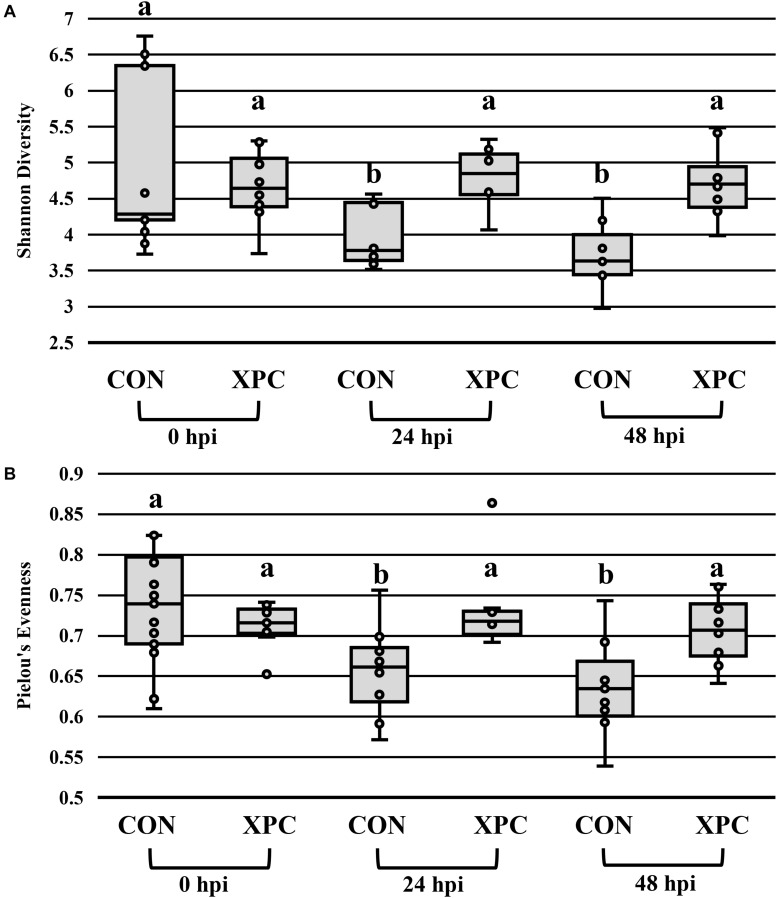
The effect of treatment on the richness **(A)** and evenness **(B)** of the microbiota of turkey ceca inoculated with *Salmonella* Typhimurium and Reading during an *in vitro* turkey cecal model with and without the supplementation of Original XPC^TM^ at 0, 24, and 48 h post-inoculation (hpi) (*P* < 0.05, *Q* < 0.05, *n* = 16, *N* = 96). Those with different superscripts (a–b) are significantly different than one another.

In congruence with the microbiological results, the microbiota results demonstrated no difference between serovars and therefore were pooled to create a protected pool (*n* = 16, *N* = 96). As such, XPC-treated ceca had a higher Shannon diversity index at 0 hpi (Figure 2A and Table 4). As time progressed throughout the *in vitro* study, the control group demonstrated a stepwise reduction in diversity through 48 hpi. However, the XPC-treated groups exhibited an increased richness that stabilized during the trial. This effect was mirrored in Pielou’s evenness, where evenness declined with each passing timepoint in the CON group while evenness narrowed initially in the XPC group and then stabilized with greater evenness at 48 h (Figure 2B and Table 4).

**TABLE 4:** The effect of treatment on the richness and evenness of the turkey cecal microbiota at 0, 24, and 48 h post-inoculation (hpi) of *Salmonella* Typhimurium and Reading during an *in vitro* turkey cecal model with and without the supplementation of Original XPC^TM^ (*P* < 0.05, *n* = 16, *N* = 96).

Alpha diversity^1,2^		Shannon diversity index			Pielou’s evenness index		
		
Group 1	Group 2	*H*^3^	*p*-value	*q*-value	*H*	*p*-value	*q*-value
0 CON	0 XPC	0.520	0.471	0.543	1.111	0.292	0.438
0 CON	24 CON	**7.089**	**0.008**	**0.015**	**7.388**	**0.007**	**0.020**
0 CON	24 XPC	1.357	0.244	0.366	0.308	0.579	0.639
0 CON	48 CON	**10.952**	**0.001**	**0.004**	**8.364**	**0.004**	**0.020**
0 CON	48 XPC	0.889	0.346	0.471	1.772	0.183	0.305
0 XPC	24 CON	**7.000**	**0.008**	**0.015**	**6.606**	**0.010**	**0.022**
0 XPC	24 XPC	0.691	0.406	0.507	0.006	0.940	0.940
0 XPC	48 CON	**10.140**	**0.001**	**0.004**	**7.707**	**0.006**	**0.020**
0 XPC	48 XPC	0.000	1.000	1.000	0.280	0.597	0.639
24 CON	24 XPC	**11.571**	**0.001**	**0.004**	**8.691**	**0.003**	**0.020**
24 CON	48 CON	1.707	0.191	0.319	0.807	0.369	0.461
24 CON	48 XPC	**8.691**	**0.003**	**0.008**	**5.491**	**0.019**	**0.036**
24 XPC	48 CON	**11.760**	**0.001**	**0.004**	**8.167**	**0.004**	**0.020**
24 XPC	48 XPC	0.206	0.650	0.697	0.823	0.364	0.461
48 CON	48 XPC	**10.667**	**0.001**	**0.004**	**6.827**	**0.009**	**0.022**

*^1^Alpha diversity metrics were analyzed using pairwise comparisons in Kruskal–Wallis.*

*^2^Bolded values are those with significant *p* and *q* values.*

*^3^H-value: test statistic for Kruskal–Wallis.*

**FIGURE 3:**
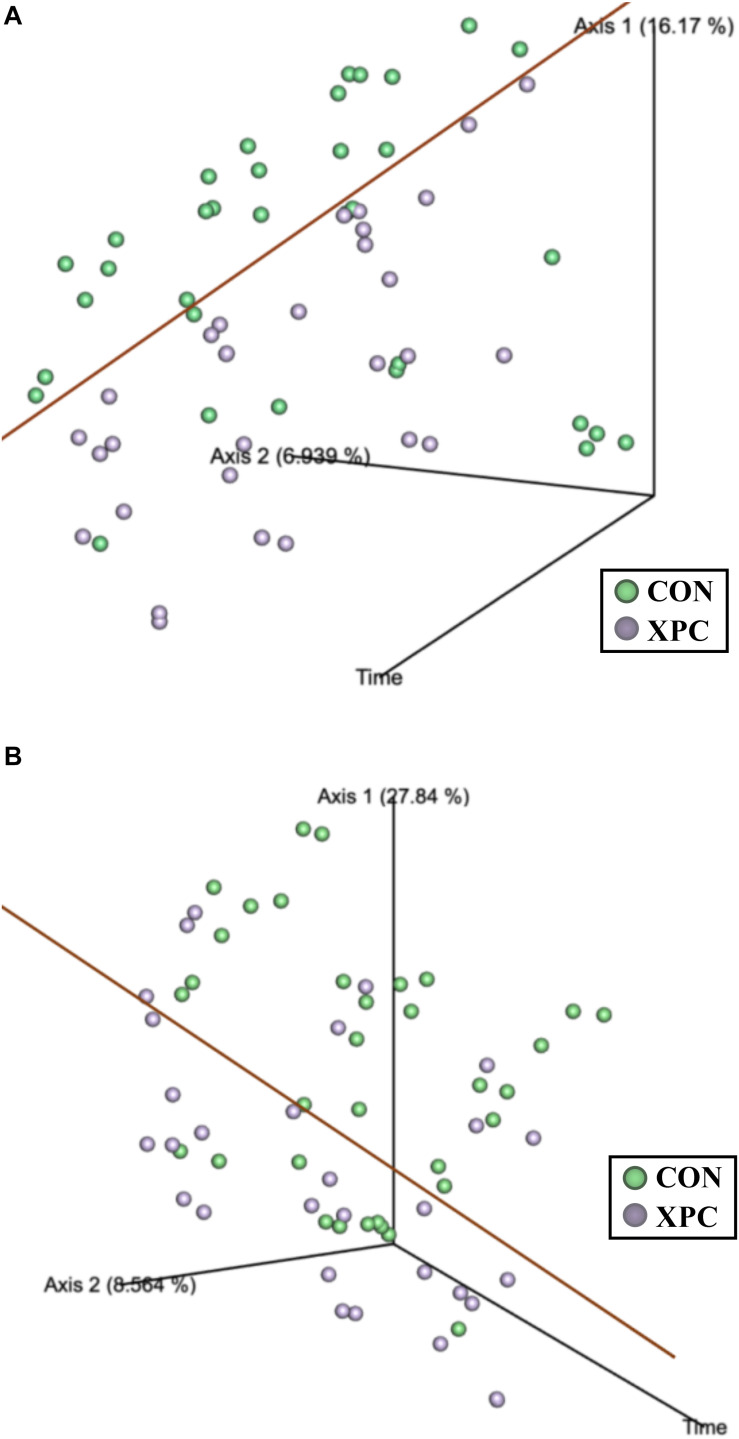
The effect of treatment on of the Jaccard dissimilarity **(A)** and weighted UniFrac distance **(B)** matrices of the turkey cecal microbiota at 0, 24, and 48 h post-inoculation (hpi) of *Salmonella* Typhimurium and Reading during an *in vitro* turkey cecal model with and without the supplementation of Original XPC^TM^ (*P* < 0.05, *Q* < 0.05, *n* = 16, *N* = 96). The green data points represent the control cecal cultures, whereas the purple data points represent the XPC treated turkey cecal cultures.

Beta diversity describes the binary differences in community structure and the differences in variation each treatment has for the quantity of OTUs present. The metric does not specify how a sample is compositionally dissimilar, but it does delineate that differences exist. The ANOSIM indicated that the Jaccard dissimilarity matrix was different by time and treatment (Table 5). Therefore, the effect of time was plotted as a continuous variable on a PCoA plot, and the individual treatments were visualized (Figure 3). The effect of treatment was significant, with differences becoming more profound over time (*Q* < 0.05; Figure 3A and Table 5). The XPC-treated groups were more diverse by the Jaccard dissimilarity matrix. Weighted Unifrac distance matrix was used to determine how the phylogenetic weight of the abundance OTUs contributed to the analysis. Like the Jaccard dissimilarity matrix that is based on presence/absence, the weighted Unifrac distance matrix is quantitative. However, besides just binary differences in diversity weighed by abundance, the weighted Unifrac distance matrix incorporates the phylogenetic branch length-weight into the analysis (Figure 3B and Table 5). In short, the use of XPC resulted in the stability of beta diversity over time (*Q* < 0.05). Irrespective of Alpha or Beta diversity index, there was no difference between the two serovars.

**TABLE 5:** The effect of treatment on the Jaccard dissimilarity and weighted UniFrac distance matrices of the turkey cecal microbiota at 0, 24, and 48 h post-inoculation (hpi) of *Salmonella* Typhimurium and Reading during an *in vitro* turkey cecal model with and without the supplementation of Original XPC^TM^ (*P* < 0.05, *n* = 16, *N* = 96).

ANOSIM beta diversity^1,2^		Jaccard dissimilarity matrix			Weighed Unifrac distance matrix		
		
Group 1	Group 2	*R*^3^	*p*-value	*q*-value	*R*	*p*-value	*q*-value
0 CON	0 XPC	0.013	0.340	0.425	0.048	0.188	0.217
0 CON	24 CON	0.010	0.332	0.425	0.136	0.049	0.074
0 CON	24 XPC	0.070	0.144	0.240	0.129	0.061	0.083
0 CON	48 CON	0.000	0.391	0.451	**0.254**	**0.015**	**0.028**
0 CON	48 XPC	0.126	0.056	0.107	**0.193**	**0.018**	**0.030**
0 XPC	24 CON	**0.398**	**0.003**	**0.011**	**0.407**	**0.001**	**0.004**
0 XPC	24 XPC	0.025	0.269	0.404	0.079	0.115	0.144
0 XPC	48 CON	**0.482**	**0.001**	**0.010**	**0.595**	**0.001**	**0.004**
0 XPC	48 XPC	0.131	0.057	0.107	**0.216**	**0.008**	**0.017**
24 CON	24 XPC	**0.271**	**0.006**	**0.015**	**0.296**	**0.002**	**0.005**
24 CON	48 CON	−0.085	0.910	0.910	−0.026	0.568	0.609
24 CON	48 XPC	**0.427**	**0.002**	**0.010**	**0.326**	**0.001**	**0.004**
24 XPC	48 CON	**0.305**	**0.006**	**0.015**	**0.370**	**0.001**	**0.004**
24 XPC	48 XPC	−0.029	0.618	0.662	−0.034	0.653	0.653
48 CON	48 XPC	**0.431**	**0.002**	**0.010**	**0.334**	**0.002**	**0.005**

*^1^Beta diversity metrics were analyzed using pairwise comparisons in ANOSIM.*

*^2^Bolded values are those with significant *p* and *q* values.*

*^3^*R*, the ANOSIM statistic, compares the mean of ranked dissimilarities between groups to the mean of ranked dissimilarities within groups.*

In order to ascertain the compositional differences between the treatment groups, analysis of composition of microbiomes (ANCOM) was used. ANCOM incorporates a strict false discovery rate that is capable of increasing statistical power. When compared in a pairwise fashion, the differences are pairwise by treatment. However, when comparing treatments over time, the OTUs are correlated to the treatment effects. The family level analysis was chosen as the family level dynamics are better annotated and could lend themselves to general structural changes by treatment. Additionally, as no diversity indices or plate count results indicated a difference between the two *Salmonella* serovars, all *Salmonella* results were treated the same for these analyses. As such, this brought the total number of replicates per treatment per timepoint to 16 (*n* = 16), which enhanced the statistical power of the study.

**FIGURE 4:**
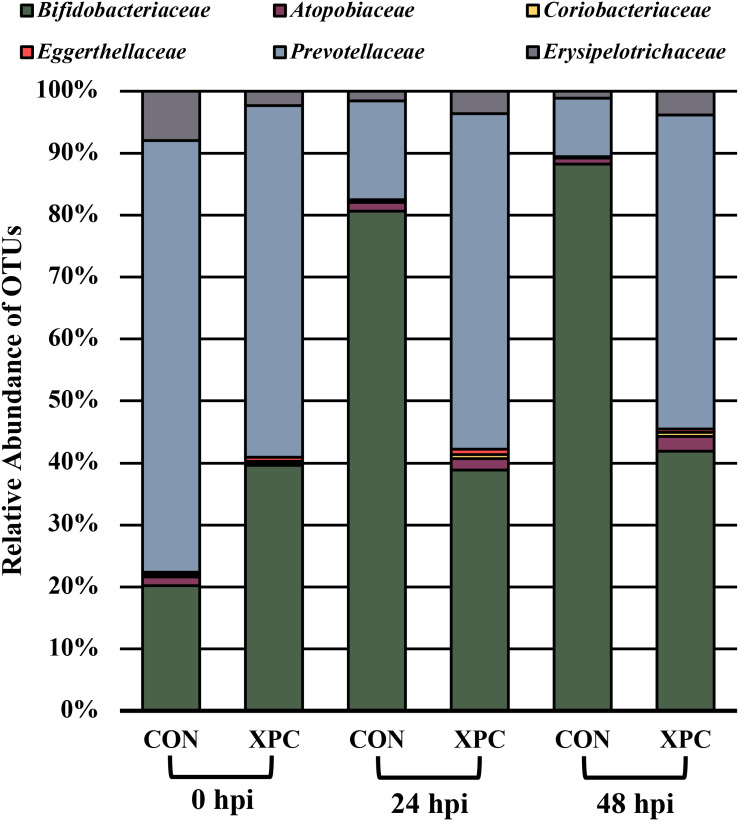
The effect of treatment on the composition of the turkey cecal microbiota at 0, 24, and 48 h post-inoculation (hpi) of *Salmonella* Typhimurium and Reading during an *in vitro* turkey cecal model with and without the supplementation of Original XPC^TM^. Sequences were compared with ANCOM at the family level (*P* < 0.05; *n* = 16; *N* = 96; *W* = 89, 76, 76, 73, 69, and 66).

Figure 4 documents the total effects of time by treatment and their associated OTUs (*P* < 0.05). At 0 hpi, XPC groups had an increase in *Bifidobacteriaceae*, *Eggerthellaceae*, and *Prevotellaceae*, and a contraction in the *Atopobiaceae*, *Coriobacteriaceae*, and *Erysipelotrichaceae* abundance of families as compared to the CON. By 24 hpi, the CON group had an increase in *Bifidobacteriaceae*. Meanwhile, the XPC group exhibited an increase in *Eggerthellaceae*, *Prevotellaceae*, *Atopobiaceae*, and *Coriobacteriaceae*. By 48 h, when Alpha and Beta diversities were at their lowest for CON, significant populations were dominated by *Bifidobacteriaceae* in the CON group. The XPC-treated group had an increase in *Eggerthellaceae*, *Prevotellaceae*, and *Coriobacteriaceae*.

**FIGURE 5:**
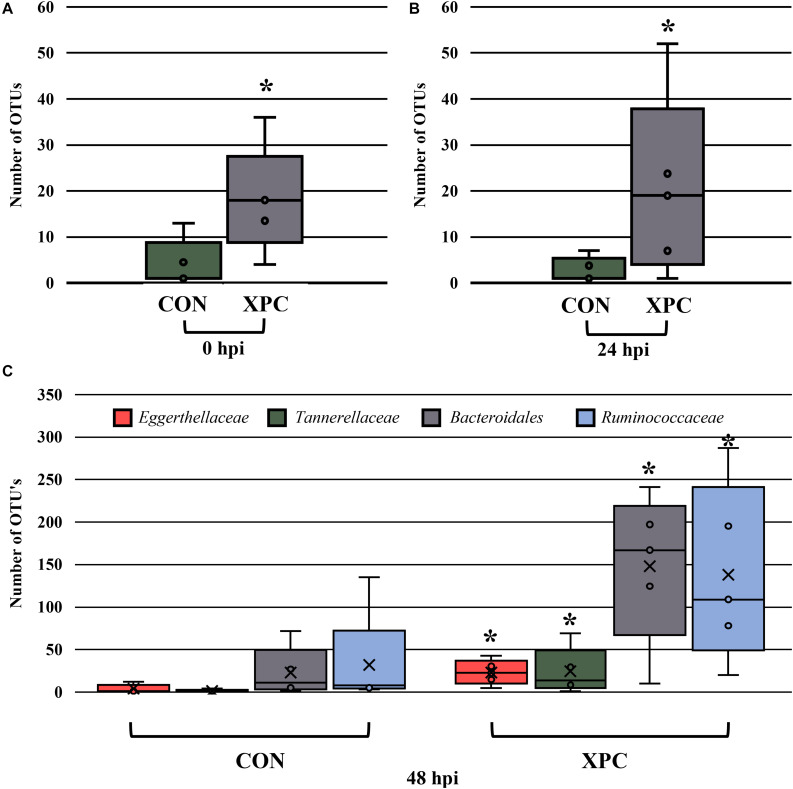
The effect of treatment on the composition of the turkey cecal microbiota at 0 **(A)**, 24 **(B)**, and 48 **(C)** h post-inoculation (hpi) of *Salmonella* Typhimurium and Reading during an *in vitro* turkey cecal model with and without the supplementation of Original XPC^TM^. Sequences were filtered by hpi and compared with ANCOM at the family level (*P* < 0.05, *Q* < 0.05, *n* = 16, *N* = 96). At 0 h, *Eggerthellaceae* was the only significant different family identified. At 24 hpi, *Coriobacteriacea* was different, and at 48 hpi, *Eggerthellaceae*, *Tannerellaceae*, *Bacteriodales*, and *Ruminococcaceae* were different. Those with an asterisk (*) have a significantly greater abundance than those without one.

In order to understand which populations had the greatest leverage at each timepoint, the sequences were filtered by hpi and compared with ANCOM. While significant fluctuations associated with treatment occurred when all comparisons were together, the only difference that emerged was *Eggerthellaceae*, where XPC had over three times more OTUs present than the CON (Figure 5A; *Q* < 0.05). By 24 hpi, *Coriobacteriacea* was higher in XPC than the control as well (Figure 5B; *Q* < 0.05). However, by 48 hpi, CON had less *Eggerthellaceae* and *Tannerellaceae*, and *Bacteriodales* (order), but an increase in *Ruminococcaceae*. Meanwhile, XPC-treated groups had an increase in *Bacteriodales* (Figure 5C).

## Discussion

In the current study, we evaluated an *in vitro* cecal model using turkey ceca. To date, it is the first study to develop and utilize this model to our knowledge. With additional validation, the *in vitro* anaerobic culture method could be valuable as a screening tool to feed amendment producers looking to understand the biology and efficacy of a particular feed component. This is especially relevant, when producers need a rapid understanding of their product’s potential impact during a public health crisis, such as what was presented in the current study with *S.* Reading. This rapid screening could help evaluate feed amendment derivatives to determine optimal dosages and combinations.

An important nuance of this study was the incorporation of *S*. Typhimurium, which was historically used in this *in vitro* model in broiler chicken ceca with significant consistency and success (Rubinelli et al., 2016, 2017; Kim et al., 2018). Ultimately, both serovars were key for the early validation of this model. This is especially true since early studies indicate that the total reduction of *Salmonella* using turkey ceca is more rapid than traditional cecal culture with chickens. Additional comparative microbiota studies directly comparing the chicken and turkey ceca will be essential if tools to work in both species are developed and attempts at comparisons are to be developed.

The use of XPC decreased the prevalence of *Salmonella* significantly throughout the study and parallels what has been observed in previous research *in vitro* and *in vivo* (Feye et al., 2016a,b, 2019; Rubinelli et al., 2016; Park et al., 2017; Roto et al., 2017; McGinnis et al., 2019). Additionally, XPC effectively reduced the pathogenic load of each *Salmonella* strain throughout the study. Interestingly, there was no difference in XPC’s ability to reduce the prevalence nor load of the two serovars preferentially. This is contrary to previously reported studies that indicate XPC may be more effective against different serovars (Feye et al., 2016a). However, the current research is confined to *S.* Typhimurium versus *S.* Reading. It should be expanded to evaluate the anti-*Salmonella* effects of XPC against all major poultry serovars of public health importance in both species as well as multiple strains within serovar to determine the range of anti-*Salmonella* effects fully. It should not be assumed this trend holds nor the chicken trends extrapolate to the turkey model as the systems are biologically distinct. Ultimately, some evidence suggests that serovars may respond differently to unique environments (Foley et al., 2013). Therefore, if recommendations regarding XPC administration are to become broadly adopted for the reduction of multiple *Salmonella* serovars, further analyses are required.

The diversity metrics in this study were also telling for the model. Previous data do not demonstrate a stepwise decrease in richness and diversity using chicken models. This difference could be explained by the limitations associated with the turkey microbiota, which tends to have a lower diversity initially. In addition, both the intestinal and cecal microbiomes of turkeys have been characterized by Wei et al. (2013) as being distinct from their chicken counterparts. Wei and colleagues attributed this demarcation to several factors, including breed, physiological, and anatomical differences, along with the slower digesta passage rate in turkeys. Ultimately, a reduction in diversity may suffer more profound and stepwise losses once the energy of the system narrows due to loss of nutrient availability. However, XPC appears to sustain diversity, which may ultimately point to changes in the metabolism of the microbiota. Likely, the narrowing is artificial, but it is demonstrative of the bottlenecks associated with turkey microbial ecology (Moxon and Kussell, 2017). More studies need to evaluate the metabolomics of the system and how sustained *in vitro* modeling impacts data interpretation.

Finally, the compositional changes associated with this model are a bit unique. Typically, in chicken models, an increase in *Ruminococcaceae* and *Archeae* occur as time progresses (Rubinelli et al., 2017; Feye et al., 2020). However, in this study, less common microorganisms persisted, which was likely due to the microbiota of the turkey. Unfortunately, the biological function of the turkey microbiota is poorly established. Therefore, speculating on the possible contribution of this consortium to the effects of XPC is premature at this time and awaits further research.

## Conclusion

XPC continues to reduce *Salmonella* as compared to the control using an *in vitro* approach. The benefits of this approach are that *Salmonella* reduction is purely microbiota mediated as the immune system and other host factors are not present. The current study was novel as it not only demonstrated the preliminary development of an *in vitro* cecal culture model using turkey ceca but demonstrated that *Salmonella* was reduced consistently using XPC regardless of serovar. However, it is of interest to screen XPC against multiple *S. enterica* serovars to determine if XPC demonstrates similar anti-*Salmonella* characteristics across all serovars as demonstrated in the current study.

Ultimately, the microbiota may become a better diagnostic tool when one considers the effects of the microbial ecology structure rather than the compositional biomarker approach. Not only is it more general, but it also does not assume specific biological meaning. In particular, models such as the one presented herein describe how the microbiota shifts independent of the immune system. From a mechanistic perspective, this is important as it indicates that XPC has greater global shifts to the microbiota, which in turn could be changing the immune system than previously described. Therefore, it may not be which organisms and when, but how the population shifts that leads to speaks to the global changes of the microbiota that impact host physiology. Understandably, this model should be further compared to an animal infection model to further delineate the anti-*Salmonella* effects of XPC.

## Data Availability Statement

The data presented in the study are deposited in the NCBI Sequence Read Archive (RSA) repository under the accession number PRJNA716729.

## Author Contributions

KF and SR conceived the trial. KF and PR conducted the benchwork for the trial. KF analyzed the data. KF and DD prepared the figures, wrote the draft, and handled edits. EO handled edits, formatting, and submissions. All authors were able to edit the publication prior to submission.

## Conflict of Interest

The authors declare that the research was conducted in the absence of any commercial or financial relationships that could be construed as a potential conflict of interest.

## Footnotes

1 https://github.com/RickeLab-UW/S.Reading-TurkeyModel.git

## References

[B1] Anderson M. J. (2001). A new method for non-parametric multivariate analysis of variance. *Austral Ecol.* 26 32–46. 10.1111/j.1442-9993.2001.01070.pp.x

[B2] Bokulich N. A. Kaehler B. D. Rideout J. R. Dillon M. Bolyen E. Knight R. (2018). Optimizing taxonomic classification of marker-gene amplicon sequences with QIIME 2’s q2-feature-classifier plugin. *Microbiome* 6:90. 10.1186/s40168-018-0470-z 29773078 PMC5956843

[B3] Bolyen E. Rideout J. R. Dillon M. R. Bokulich N. A. Abnet C. Al-Ghalith G. A. (2018). QIIME 2: reproducible, interactive, scalable, and extensible microbiome data science. *PeerJ Preprints* 6:e27295v2. 10.7287/peerj.preprints.27295v2 PMC7015180 31341288

[B4] Callahan B. J. McMurdie P. J. Rosen M. J. Han A. W. Johnson A. J. A. Holmes S. P. (2016). DADA2: high-resolution sample inference from Illumina amplicon data. *Nat. Methods* 13 581–583. 10.1038/nmeth.3869 27214047 PMC4927377

[B5] Canibe N. Jenses B. B. (2012). Fermented liquid feed-microbial and nutritional aspects and impact on enteric diseases in pigs. *Anim. Feed Sci. Technol.* 173 17–40. 10.1016/j.anifeedsci.2011.12.021

[B6] Centers for Disease Control and Prevention [CDC]. (2019). *Multidrug-Resistant Salmonella Infections Linked to Raw Turkey Products.* Available online at: https://www.cdc.gov/salmonella/reading-07-18/ (accessed August 14, 2020).

[B7] Diamond V Original XPC^TM^ (XPC) (2020). *Product Profile.* Available online at: https://www.diamondv.com/wp-content/uploads/productprofile_xpc_original.pdf (accessed December 21, 2020).

[B8] Feye K. M. Andereson K. L. Scott M. F. Henry D. L. Dorton K. L. Depenbusch B. E. (2016a). Abrogation of *Salmonella* and E. *coli* O157:H7 in feedlot cattle fed a proprietary *Saccharomyces cerevisiae* fermentation prototype. *J. Vet. Sci. Technol.* 7:350. 10.4172/2157-7579.1000350

[B9] Feye K. M. Anderson K. L. Scott M. F. McIntyre D. R. Carlson S. A. (2016b). Inhibition of virulence, antibiotic resistance, and fecal shedding of multiple-antibiotic resistant *Salmonella* Typhimurium in broilers fed Original XPC. *Poult. Sci.* 95 2901–2910. 10.3382/ps/pew254 27566726 PMC5144663

[B10] Feye K. M. Carroll J. A. Anderson K. L. Whittaker J. Schmidt-McCormack G. McIntyre D. R. (2019). *Saccharomyces cerevisiae* fermentation products that mitigate foodborne *Salmonella*. *Front. Vet. Sci.* 6:107. 10.3389/fvets.2019.00107 31024942 PMC6467977

[B11] Feye K. M. Rubinelli P. M. Chaney W. E. Pavlidis H. O. Kogut M. H. Ricke S. C. (2020). The preliminary development of an *in vitro* poultry cecal culture model to evaluate the effects of Original XPC^TM^ for the reduction of *Campylobacter jejuni* and its potential effects on the microbiota. *Front. Microbiol.* 10:3062. 10.3389/fmicb.2019.03062 32038534 PMC6990144

[B12] Foley S. L. Johnson T. J. Ricke S. C. Nayak R. Danzeisen J. (2013). *Salmonella* pathogenicity and host adaptation in chicken-associated serovars. *Microbiol. Mol. Biol. Rev.* 77 582–607. 10.1128/MMBR.00015-13 24296573 PMC3973385

[B13] Food Safety Inspection Service [FSIS] (2014). *Serotypes of Salmonella Isolates from Meat and Poultry Products January 1998 through December 2014.* Available online at: https://www.fsis.usda.gov/wps/wcm/connect/3866026a-582d-4f0e-a8ce-851b39c7390f/Salmonella-Serotype-Annual-2014.pdf?MOD=AJPERES (accessed August 20, 2020).

[B14] Gad A. H. Abo-Shama U. H. Harclerode K. K. Fakhr M. K. (2018). Prevalence, serotyping, molecular typing, and antimicrobial resistance of *Salmonella* isolated from conventional and organic retail ground poultry. *Front. Microbiol.* 9:2653. 10.3389/fmicb.2018.02653 30455678 PMC6230656

[B15] Herigstad B. Hamilton M. Heersink J. (2001). How to optimize the drop plate method for enumerating bacteria. *J. Microbiol. Methods* 44 121–129. 10.1016/S0167-7012(00)00241-4 11165341

[B16] Jett B. D. Hatter K. L. Huycke M. M. Gilmore M. S. (1997). Simplified agar plate method for quantifying viable bacteria. *Biotechniques* 23 648–650. 10.2144/97234bm22 9343684

[B17] Katoh K. Misawa K. Kuma K. Miyata T. (2002). MAFFT: a novel method for rapid multiple sequence alignment based on fast Fourier transform. *Nucleic Acids Res.* 30 3059–3066. 10.1093/nar/gkf436 12136088 PMC135756

[B18] Kim S. A. Rubinelli P. M. Park S. H. Ricke S. C. (2018). Ability of Arkansas LaKast and LaKast hybrid rice bran to reduce *Salmonella* typhimurium in chicken cecal incubations and effects on cecal microbiota. *Front. Microbiol.* 9:134. 10.3389/fmicb.2018.00134

[B19] Kozich J. J. Westcott S. L. Baxter N. T. Highlander S. K. Schloss P. D. (2013). Development of a dual-index sequencing strategy and curation pipeline for analyzing amplicon sequence data on the MiSeq Illumina sequencing platform. *Appl. Environ. Microbiol.* 79, 5112–5120. 10.1128/AEM.01043-13 23793624 PMC3753973

[B20] Kruskal W. H. Wallis W. A. (1952). Use of ranks in one-criterion variance analysis. *J. Am. Stat. Assoc.* 47 583–621. 10.1080/01621459.1952.10483441

[B21] Lozupone C. A. Hamady M. Kelley S. T. Knight R. (2007). Quantitative and qualitative beta diversity measures lead to different insights into factors that structure microbial communities. *Appl. Environ. Microbiol.* 73 1576–1585. 10.1128/AEM.01996-06 17220268 PMC1828774

[B22] Mandal S. Treuren W. V. White R. A. Eggesbø M. Knight R. Peddada S. D. (2015). Analysis of composition of microbiomes: a novel method for studying microbial composition. *Microb. Ecol. Health Dis.* 26:27663. 10.3402/mehd.v26.27663 26028277 PMC4450248

[B23] McGinnis J. Byrd J. A. Pavlidis H. O. Chaney W. E. (2019). “The effects of feeding Original XPC on reducing *Salmonella* prevalence and numbers in ceca samples and carcass rinses taken from commercial broilers,” in *Proceedings of the International Association for Food Protection Annual Meeting, July 2019*, Louisville, KY, 3–222.

[B24] Moxon R. Kussell E. (2017). The impact of bottlenecks on microbial survival, adaptation, and phenotypic switching in host-pathogen interactions. *Evolution* 71 2803–2816. 10.1111/evo.13370 28983912 PMC5722657

[B25] Naghili H. Tajik H. Mardani K. Razavi Rouhani S. M. Ehsani A. Zare P. (2013). Validation of drop plate technique for bacterial enumeration by parametric and nonparametric tests. *Vet. Res. Forum* 4 179–183. 25653794 PMC4312378

[B26] National Research Council [NRC] (1994). *Nutrient Requirements of Poultry: Ninth Revised Edition.* Washington, DC: The National Academies Press. 10.17226/2114

[B27] Nelson J. R. Sobotik E. B. Athrey G. Archer G. S. (2020). Effects of supplementing yeast fermentate in the feed or drinking water on stress susceptibility, plasma chemistry, cytokine levels, antioxidant status, and stress-and immune-related gene expression of broiler chickens. *Poult. Sci.* 99 3312–3318. 10.1016/j.psj.2020.03.037 32616224 PMC7597835

[B28] Park S. H. Kim S. A. Lee S. I. Rubinelli P. M. Roto S. M. Pavlidis H. O. (2017). Original XPC^TM^ effect on *Salmonella* Typhimurium and cecal microbiota from three different ages of birds when incubated in an anaerobic *in vitro* culture system. *Front. Microbiol.* 8:1070. 10.3389/fmicb.2017.01070 28659891 PMC5468444

[B29] Poeker S. A. Lacroix C. de Wouters T. Spalinger M. R. Scharl M. Geirnaert A. (2019). Stepwise development of an in vitro continuous fermentation model for the murine caecal microbiota. *Front. Microbiol.* 10:1166. 10.3389/fmicb.2019.01166 31191488 PMC6548829

[B30] Public Health Agency of Canada [PHAC] (2021). *Public Health Notice — Outbreak of Salmonella Illnesses Linked to Raw Turkey and Raw Chicken.* Available online at: https://www.canada.ca/en/public-health/services/public-health-notices/2018/outbreak-salmonella-illnesses-raw-turkey-raw-chicken.html (accessed March 3, 2021).

[B31] Roto S. M. Park S. H. Lee S. I. Kaldhone P. Pavlidis H. O. Frankenbach S. B. (2017). Effects of feeding Original XPC(to broilers with a live coccidiosis-vaccine under industry conditions: Part 1. Growth performance and Salmonella inhibition. *Poult. Sci.* 96 1831–1837. 10.3382/ps/pew445 28340000

[B32] Rubinelli P. M. Roto S. M. Kim S. Park S. Pavlidis H. O. McIntyre D. (2016). Reduction of *Salmonella* Typhimurium by fermentation metabolites of Diamond V Original XPC in an in vitro anaerobic mixed chicken cecal culture. *Front. Vet. Sci.* 3:83. 10.3389/fvets.2016.00083 27695699 PMC5025443

[B33] Rubinelli P. R. Kim S. A. Park S. H. Roto S. M. Nealon N. J. Ryan E. P. (2017). Differential effects of rice bran cultivars to limit *Salmonella* Typhimurium in chicken cecal in vitro incubations and impact on the cecal microbiome and metabolome. *PLoS ONE* 12:e0185002. 10.1371/journal.pone.0185002 28937988 PMC5609742

[B34] Schlosser W. Hogue A. Ebel E. Rose B. Umholtz R. Ferris K. (2000). Analysis of *Salmonella* serotypes from selected carcasses and raw ground products sampled prior to the implementation of the Pathogen Reduction: hazard analysis and critical control point final rule in the US. *Int. J. Food Microbiol.* 58 107–111. 10.1016/s0168-1605(00)00293-2 10898467

[B35] Wei S. Morrison M. Yu Z. (2013). Bacterial census of poultry intestinal microbiome. *Poult. Sci.* 92 671–683. 10.3382/ps.2012-02822 23436518

[B36] Zhao S. McDermott S. Friedman J. Abbot S. Ayers A. Gleen E. (2006). Antimicrobial resistance and genetic relatedness among *Salmonella* from retail foods of animal origin: NARMS retail meat surveillance. *Foodborne Pathog. Dis.* 3 106–117. 10.1089/fpd.2006.3.106 16602986

