# Supplementary material for: Yeast Fermentate-Mediated Reduction of Salmonella Reading and Typhimurium in an in vitro Turkey Cecal Culture Model
Source: Front Microbiol. 2021 Apr 15;12:645301. doi: 10.3389/fmicb.2021.645301 (PMC8081899; doi:10.3389/fmicb.2021.645301)
Supplement: Supplementary file 1 [file Table_1.DOCX]

**Supplemental Tables**

**Supplemental Table 1**. Means, Standard Error the Means (SEMs), and P values of the effect and interactions of Treatment, Serovar, and Time on the prevalence of *Salmonella* with Time as a repeated measure.

| **Time (h)^1,2^** | ***Salmonella* Serovar^2,3^** | **Treatment^2,4^** | **Mean** | **±** | **SEM** |
| --- | --- | --- | --- | --- | --- |
| 0 | *S.* Typhimurium | CON | 100.00 | ± | 0.000 |
|  |  | XPC | 100.00 | ± | 0.000 |
|  | *S*. Reading | CON | 100.00 | ± | 0.000 |
|  |  | XPC | 100.00 | ± | 0.000 |
| 24 | *S*. Typhimurium | CON | 100.00 | ± | 0.000 |
|  |  | XPC | 90.00 | ± | 9.487 |
|  | *S*. Reading | CON | 90.00 | ± | 9.487 |
|  |  | XPC | 60.00 | ± | 15.492 |
| 48 | *S.* Typhimurium | CON | 70.00 | ± | 14.145 |
|  |  | XPC | 0.00 | ± | 0.000 |
|  | *S.* Reading | CON | 70.00 | ± | 14.491 |
|  |  | XPC | 30.00 | ± | 14.491 |

^1^Effect of time; P < 0.0001, N = 120, n = 40, k = 3.

^2^Interaction of Time, *Salmonella* Serovar, and Treatment; P = 0.2106, N = 120, n = 10, k = 12.

^3^Effect of *Salmonella* Serovar; P = 0.7735, N = 120, n = 60, k = 2.

^4^Effect of Treatment; P < 0.0001, N = 60, k = 2.

**Supplemental Table 2.** There was no effect of serovar (P = 0.7735), *Salmonella* Typhimurium and *S*. Reading, or the interaction of serovar × time × treatment (P = 0.2106) on the prevalence of *Salmonella.* Serovars were separated to look at within differences. The effect of time and treatment and their subsequent interaction on the prevalence of *Salmonella* when *Salmonella* Typhimurium or *S*. Reading were inoculated into an *in vitro* turkey cecal model.

| ***Salmonella* Typhimurium Prevalence** | | | | | | |
| --- | --- | --- | --- | --- | --- | --- |
| **Time** |  | **P-value** | | **<** | **0.0001** | |
| Variable |  | Mean |  | ± | SEM^1^ | |
| 0 |  | 100.00 | ^a^ | ± | 0.00 | |
| 24 |  | 95.00 | ^a^ | ± | 0.00 | |
| 48 |  | 35.00 | ^b^ | ± | 9.75 | |
|  |  |  |  |  |  | |
| **Treatment** |  | **P-value** | | **<** | **0.0001** | |
| Variable |  | Mean |  |  | SEM^1^ | |
| CON |  | 90.00 | ^a^ | ± | 16.43 | |
| XPC |  | 63.33 | ^b^ | ± | 26.39 | |
|  |  |  |  |  |  | |
| **Time*Treatment** | | **P-value** | | **<** | **0.0001** | |
| Variable |  | Mean |  |  | SEM^1^ | |
| 0 | CON | 100.00 | ^a^ | ± | 0.00 | |
|  | XPC | 100.00 | ^a^ | ± | 0.00 | |
| 24 | CON | 100.00 | ^a^ | ± | 0.00 | |
|  | XPC | 90.00 | ^ab^ | ± | 9.49 | |
| 48 | CON | 70.00 | ^b^ | ± | 14.49 | |
|  | XPC | 0.00 | ^c^ | ± | 0.00 | |
|  | | | | | | |
| ***Salmonella* Reading Prevalence** | | | | | | |
| **Time** |  | **P-value** | | **=** | | **0.0004** |
| Variable |  | Mean |  |  | | SEM^2^ |
| 0 |  | 100.00 | ^a^ | ± | | 0.00 |
| 24 |  | 75.00 | ^b^ | ± | | 9.75 |
| 48 |  | 50.00 | ^c^ | ± | | 19.36 |
|  |  |  |  |  | |  |
| **Treatment** |  | **P-value** | | **=** | | **0.0187** |
| Variable |  | Mean |  |  | | SEM^2^ |
| CON |  | 86.67 | ^a^ | ± | | 18.62 |
| XPC |  | 63.33 | ^b^ | ± | | 26.39 |
|  |  |  |  |  | |  |
| **Time*Treatment** | | **P-value** | | **=** | | **0.2195** |
| Variable |  | Mean |  |  | | SEM^2^ |
| 0 | CON | 100.00 |  | ± | | 0.00 |
|  | XPC | 100.00 |  | ± | | 0.00 |
| 24 | CON | 90.00 |  | ± | | 9.49 |
|  | XPC | 60.00 |  | ± | | 15.49 |
| 48 | CON | 70.00 |  | ± | | 14.49 |
|  | XPC | 30.00 |  | ± | | 14.49 |

^1^*Salmonella* Typhimurium Pooled SEM = 5.27, 4.30, 7.45; N = 60, 60, 60; n = 20, 30, 10; k = 3, 2, 6

^2^*Salmonella* Reading Pooled SEM = 8.33, 6.80, 11.79; N = 60, 60, 60; n = 20, 30, 10; k = 3, 2, 6

**Supplemental Table 3.** Means, Standard Error the Means (SEMs), and P values of the effect and interactions of Treatment, Serovar, and Time on the total load of *Salmonella* with Time as a repeated measure.

| **Time (h)^1,2^** | ***Salmonella* Serovar^2,3^** | **Treatment^2,4^** | **Mean** | **±** | **SEM** |
| --- | --- | --- | --- | --- | --- |
| 0 | *S.* Typhimurium | CON | 7.254 | ± | 0.054 |
|  |  | XPC | 7.245 | ± | 0.038 |
|  | *S*. Reading | CON | 7.409 | ± | 0.042 |
|  |  | XPC | 7.496 | ± | 0.030 |
| 24 | *S*. Typhimurium | CON | 4.201 | ± | 0.386 |
|  |  | XPC | 2.445 | ± | 0.331 |
|  | *S*. Reading | CON | 4.254 | ± | 0.547 |
|  |  | XPC | 1.717 | ± | 0.471 |
| 48 | *S.* Typhimurium | CON | 2.158 | ± | 0.616 |
|  |  | XPC | 0.000 | ± | 0.000 |
|  | *S.* Reading | CON | 2.065 | ± | 0.539 |
|  |  | XPC | 0.710 | ± | 0.371 |

^1^Effect of time; P < 0.0001, N = 120, n = 40, k = 3.

^2^Interaction of Time, *Salmonella* Serovar, and Treatment; P = 0.2891, N = 120, n = 10, k = 12.

^3^Effect of *Salmonella* Serovar; P = 0.7779, N = 120, n = 60, k = 2.

^4^Effect of Treatment; P < 0.0001, N = 60, k = 2.

**Supplemental Table 4**. There was no significant effect of serovar (P = 0.7779), *Salmonella* Typhimurium and *S*. Reading, or the interaction of serovar × time × treatment (P = 0.2891) on the load of *Salmonella* . Serovars were separated to look at within serovar differences. The effect of time and treatment and their subsequent interaction on the load of *Salmonella* when *Salmonella* Typhimurium or *S*. Reading were inoculated into an *in vitro* turkey cecal model.

| ***Salmonella* Typhimurium (Log_10_ CFU/mL)** | | | | | | |
| --- | --- | --- | --- | --- | --- | --- |
| **Time** |  | **P-value** | | **<** | | **0.0001** |
| Variable |  | Mean |  |  | | SEM |
| 0 |  | 7.25 | ^a^ | ± | | 0.032 |
| 24 |  | 3.32 | ^b^ | ± | | 0.319 |
| 48 |  | 1.08 | ^c^ | ± | | 0.389 |
|  |  |  |  |  | |  |
| **Treatment** |  | **P-value** | | **<** | | **0.0001** |
| Variable |  | Mean |  |  | | SEM |
| CON |  | 4.54 | ^a^ | ± | | 0.454 |
| XPC |  | 3.23 | ^b^ | ± | | 0.569 |
|  |  |  |  |  | |  |
| **Time*Treatment** | | **P-value** | | **=** | | **0.0041** |
| Variable |  | Mean |  |  | | SEM |
| 0 | CON | 7.25 | ^a^ | ± | | 0.054 |
|  | XPC | 7.25 | ^a^ | ± | | 0.038 |
| 24 | CON | 4.20 | ^b^ | ± | | 0.386 |
|  | XPC | 2.45 | ^c^ | ± | | 0.331 |
| 48 | CON | 2.16 | ^c^ | ± | | 0.616 |
|  | XPC | 0.00 | ^d^ | ± | | 0.000 |
|  | | | | | | |
| ***Salmonella* Reading (Log_10_ CFU/mL)** | | | | | | |
| **Time** |  | **P-value** | | **<** | **0.0001** | |
| Variable |  | Mean |  | ± | SEM | |
| 0 |  | 7.45 | ^a^ | ± | 0.027 | |
| 24 |  | 2.99 | ^b^ | ± | 0.456 | |
| 48 |  | 1.39 | ^c^ | ± | 0.354 | |
|  |  |  |  |  |  | |
| **Treatment** |  | **P-value** | | **=** | **0.0003** | |
| Variable |  | Mean |  |  | SEM | |
| CON |  | 4.58 | ^a^ | ± | 0.477 | |
| XPC |  | 3.31 | ^b^ | ± | 0.589 | |
|  |  |  |  |  |  | |
| **Time*Treatment** | | **P-value** | | **=** | **0.0070** | |
| Variable |  | Mean |  |  | SEM | |
| 0 | CON | 7.41 | ^a^ | ± | 0.043 | |
|  | XPC | 7.50 | ^a^ | ± | 0.030 | |
| 24 | CON | 4.25 | ^b^ | ± | 0.547 | |
|  | XPC | 1.72 | ^cd^ | ± | 0.471 | |
| 48 | CON | 2.07 | ^c^ | ± | 0.539 | |
|  | XPC | 0.71 | ^d^ | ± | 0.371 | |

^1^*Salmonella* Typhimurium Pooled SEM = 0.23, 0.19, 0.33; N = 60, 60, 60; n = 20, 30, 10; k = 3, 2, 6

^2^*Salmonella* Reading Pooled SEM = 0.28, 0.23, 0.40; N = 60, 60, 60; n = 20, 30, 10; k = 3, 2, 6
